# A comparison of endoscopic submucosal dissection (ESD) and radical surgery for early gastric cancer: a retrospective study

**DOI:** 10.1186/s12957-015-0724-1

**Published:** 2015-11-04

**Authors:** Wen-chong Song, Xiu-li Qiao, Xiao-zhong Gao

**Affiliations:** Gastroenterology Division, Weihai Municipal Hospital, Weihai, 264200 People’s Republic of China

**Keywords:** Endoscopic submucosal dissection, Radical operation, Early gastric cancer

## Abstract

**Background:**

Endoscopic submucosal dissection (ESD) has become one of the mainstays of treatment for early gastric cancer (EGC). Radical surgery is also a classical treatment method for EGC. There have been no systematic clinical studies of the curative effects and adverse events associated with ESD vs. radical surgery for EGC. This study investigated the therapeutic efficacy and safety of ESD and radical surgery for EGC.

**Methods:**

Twenty-nine patients with EGC underwent ESD, and 59 underwent radical surgery at Weihai Municipal Hospital. The pathological characteristics, postoperative outcomes, hospital course, morbidity and mortality were retrospectively compared between the two groups.

**Results:**

The oncological clearance was 93.1 % (27/29) in the ESD group. Postoperative delayed haemorrhage occurred in two patients. The hospital stay ranged from 10 to 23 days, and the average stay was 14.3 ± 3.7 days. The patients were followed-up for 1 to 5 years, with a mean follow-up of 26.9 ± 8.5 months. Regular endoscopic examinations showed that the wound had healed with no cancer recurrence in all of the patients. In the radical surgery group, the oncological clearance was 100 % (59/59). The hospital stay ranged from 11 to 55 days, and the average stay was 21.7 ± 9.3 days. The patients were followed-up for 1 to 3.7 years, with a mean follow-up of 22.3 ± 9.4 months. Nine patients developed complications, including acute postoperative adhesive ileus (1/59) and symptomatic residual gastritis (3/59). These complications were improved by an additional operation, drainage, gastrointestinal decompression and comprehensive therapy.

**Conclusions:**

ESD achieved similar efficacy and had many advantages compared with radical surgery for the treatment of EGC.

## Background

Gastric cancer is one of the most common malignant tumours and is the second leading cause of cancer death worldwide [1]. In China, the mortality rate of gastric cancer is the highest of all cancers and accounts for 23.2 % of the total mortality caused by malignancy [2]. Despite advances in the diagnosis and treatment of gastric cancer, the prognosis of this type of tumour remains poor because the vast majority of patients are diagnosed at an advanced stage. Therefore, it is necessary to identify ways to diagnose and treat gastric cancer at an earlier stage [3].

Surgery is the conventional and most definite loco-regional treatment for early gastric cancer (EGC) [4]. However, surgery for gastric cancer is associated with significant perioperative morbidities. Because of postoperative anatomical and physiological alterations, some patients suffer from refractory complications, such as serious oesophageal acid reflux, bile reflux gastritis, remnant gastric cancer and difficult defecation [5–9]. Hence, surgery is also associated with a significant decrease in the quality of life (QOL) of patients [5–9]. In recent years, endoscopic submucosal dissection (ESD) has been advocated as a new approach for EGC treatment. It has many advantages, such as its minimally invasive nature, good curative effect, shorter average hospitalisation time and the fact that the normal physiological structure of the gastrointestinal tract is retained [10–13]. Although various complications have been described, including bleeding, perforation, stenosis, aspiration pneumonia, phlegmonous gastritis, mediastinal emphysema, residual tumour or recurrence, the ESD-related complication rates have been relatively low [14]. However, there have not been any systematic clinical studies of the curative effects, complications, recurrence rates, and QOL of patients who underwent ESD compared with those who underwent a radical operation for the treatment of EGC. Therefore, we conducted a retrospective cohort study comparing radical surgery with ESD for the treatment of EGC.

In recent years, endoscopic submucosal dissection (ESD) has become widely accepted as a less invasive treatment for early gastric cancer (EGC) [10–13]. Radical surgery is the classical treatment for EGC [4]. Twenty-nine patients with EGC underwent ESD and 59 underwent radical surgery for EGC from August 2007 to March 2012 at Weihai Municipal Hospital. The follow-up period was at least 1 year for all patients. The pathological characteristics, postoperative outcomes, hospital course, morbidity and mortality were retrospectively compared.

## Methods

### Patients

We retrospectively reviewed the records of the patients with EGC who underwent ESD or radical surgery who were admitted to Weihai Municipal Hospital between August 2007 and March 2012. The patients admitted during this period were divided into a conventional radical surgery group and an ESD group according to the therapeutic method used. The patient background data, complete resection (CR) rate, length of the operation, blood loss, perforation rate, curative effect, long-term complications, recurrence rate and QOL were compared between the groups. Patients with severe underlying disease, such as heart disease, respiratory disease, liver disease or a bleeding tendency, were excluded from the indications for both ESD and radical surgery at our institute; however, no patients had severe underlying disease in this study. If patients had taken drugs to prevent clotting, such as aspirin or warfarin, the ESD or radical operation was performed after a fixed term of drug discontinuance.

All patients fulfilled the following criteria: the tumour was limited to the mucosal or submucosal layers without distant organ or any lymph node involvement detected using a combined examination by endoscopic ultrasonography (EUS), PET scanning and abdominal computed tomography (CT); in addition, the diameter of the lesion was not more than 50 mm. The performance status (PS) of each patient was less than 2 on the Eastern Cooperative Oncology Group (ECOG) scale. All patients who underwent ESD or radical resection were discussed at an MDT meeting (Gastrointestinal Surgery, Gastroenterology, Radiology, Interventional Radiology, and Oncology departments). Finally, 29 patients with EGC who underwent ESD and 59 patients with EGC who underwent a radical operation from August 2009 to March 2012 at Weihai Municipal Hospital were included in the study.

After being enrolled, the ESD or radical operation was performed with the consent of these patients after they were hospitalised and informed of the risks and benefits of the treatment methods. Written and informed consent was obtained to publish and to report individual patient data from all patients, and the study was conducted upon approval by the Ethics Committee of Weihai Municipal Hospital. More than 100 ESD procedures had been performed before starting the study.

### Surgical procedures

#### ESD technique

The margins of the lesion were delineated before ESD using narrow-band imaging (NBI) and 0.4 % indigo carmine dye spraying and were marked with argon plasma coagulation (APC). The submucosa was raised through submucosal injection. After the injection, a circumferential incision was made using a HOOK-knife. Repeated injections were performed to lift the lesion, and the lesion was cut using an IT knife. The ulcer bed after the en-bloc resection was treated by hot biopsy forceps and APC. Some wounds were closed with metal clips. All lesions were sent for a pathological examination.

### Technique used for radical surgery

#### Radical distal gastrectomy with D2 dissection

First, a routine exploration of the abdominal cavity was performed. The left greater omentum was then dissected from the transverse colon and the lymph nodes along the left gastroepiploic vessels (No. 4sb). Then, the right omentum and the lymph nodes were dissected along the right gastroepiploic vessels (No. 4d). The superior mesenteric vein, Henle's trunk, right colic vein, and right gastroepiploic vein and artery were exposed to allow for dissection of lymph nodes No. 6 and 14v. Then, the suprapyloric nodes (No. 5) and the nodes along the proper hepatic artery (No. 12a) were dissected, followed by dissection of the nodes along the left gastric artery (No. 7) and nodes along the celiac artery (No. 9). Subsequently, the nodes along the common hepatic artery (No. 8a) and the proximal splenic artery (No. 11p) were dissected. The duodenum was transected just distal to the pyloric ring using a 45-mm Endo-GIA stapler. This was followed by dissection of the lesser curvature in the remnant stomach, with dissection of the right cardial nodes (No. 1) and the nodes along the lesser curvature (No. 3). Subsequently, the stomach on the upper third above the tumour was transected. Finally, an upper midline incision (approximately 5 cm) was made, the gastrectomy was performed and the gastrointestinal continuity was restored using a Billroth I or II anastomosis through this incision. All lesions were sent for a pathological examination.

#### Radical proximal gastrectomy with D2 dissection

The D2 lymphadenectomy was performed in the same manner as described above. The gastrointestinal continuity was reconstructed in the oesophagogastric anastomosis through this incision.

### Histological assessment

A pathologist evaluated the specimens obtained by ESD or radical surgery, paying special attention to the depth of tumour invasion and the lateral and deep margins of the excision. The resected specimens were cut into 2-mm slices and evaluated to determine whether cancerous glands were present at the margin of each slice.

### Definitions of complete and incomplete resection

When the margins were definitely free of tumorous glands after the ESD procedure, the resection was considered to be complete. Multi-fragment resections were incomplete when tumorous glands were present in one or more fragments histologically. If the lateral margin of the lesion could not be evaluated histologically because of the effects of the electrosurgical current or mechanical damage, the resection was considered incomplete.

The assessment of complete and incomplete resections after radical surgery was similar to that after ESD. When the margin of resected specimens, the distal resected omentum, and the distal lymph nodes were definitely free of tumorous glands, the resection was considered to be complete. Resections were defined as incomplete when tumorous glands were present in one or more the distal resected omentum and/or lymph nodes histologically.

#### Follow-up

The follow-up gastroscopy and observation were performed at 1, 3, 6, and 12 months postoperatively. The Chinese versions of the European Organization for Research and Treatment of Cancer (EORTC) Quality of Life Questionnaire Core 30 (QLQ-C30) and a gastric cancer-specific module, the EORTC QLQ-STO22, were used to assess the QOL. The scores were calculated by investigating 15 scales from the EORTC QLQ-C30 and nine scales from the EORTC QLQ-STO22. In the EORTC QLQC30, the QOL is higher when the general health and functional scale scores are higher, and the QOL is lower when the symptom scale score is higher. In the EORTC QLQ-STO22, the QOL is lower when the scores for each category are higher [15–17]. The follow-up deadline was March 2013. All patients underwent gastroscopy, and pathological biopsies were collected to assess the patients for residual or recurrent tumours. The recent and long-term complications and their management were investigated. The shortest follow-up period was 1 year, and the longest was 5 years.

### Statistical analysis

The values are expressed as the means ± SD. The statistical analysis was performed using the unpaired Students *t* test, ANCOVA and the chi-square test. A *P* value <0.05 was considered significant.

## Results

### Patient backgrounds

A total of 88 patients participated in the study. The final disease stage was classified according to the 6th Union for International Cancer Control (UICC) [18]. Twenty-nine patients with EGC underwent ESD, and 59 underwent a radical operation. The median age in the ESD group [65.3 ± 7.5 years (46–83 years)] was significantly older than that in the radical operation group [45.8 ± 6.7 years (24–80 years)]. There were 14 women in the ESD group and 21 women in the radical operation group. The location and gross tumour type were not significantly different between the two groups. Although the median size of the lesion was 2.7 ± 1.9 cm in the ESD group and 3.5 ± 1.6 cm in the radical operation group, there was no significant difference between the two groups (Tables 1 and 2). Adjuvant chemotherapy was not administered to any of the patients.

**Table 1:** The tumour characteristics of the patients in the ESD and radical operation groups

	ESD group	Radical operation group	*P*
*n*	29	59	
Location of the tumour (%)			
Posterior wall	6 (20.7)	11 (18.6)	NS
Anterior wall	7 (24.1)	19 (32.2)	NS
Lesser curvature	9 (31.1)	16 (27.1)	NS
Greater curvature	7 (24.1)	13 (22)	NS
Gross type of the tumour (%)			
Superficial depressed type	17 (58.6)	33 (55.9)	NS
Superficial elevated type	12 (41.4)	26 (44.1)	NS

**Table 2:** The characteristics and clinical data of the patients in the ESD and radical operation groups

	ESD group (tumour size, 0.8–5.0 cm)	Radical operation group (tumour size, 1.1–5.7 cm)	*P*
*N*	29	59	
Mean age (years)	65.3 ± 7.5	45.8 ± 6.7	<0.05
Mean size of the lesion (cm)	2.7 ± 1.9	3.5 ± 1.6	NS
Frequency of underlying cardiopulmonary disease (%)	17.2	5.1	<0.01
Frequency of anticoagulant therapy (%)	13.8	1.7	<0.01
Length of operation (min)	53 ± 7.9	78 ± 11.4	<0.05
Depth of invasion (mucosa: submucosa:muscularis propria)	27:02:00	47:12:00	<0.05
Hospitalisation (d)	14.3 ± 3.7	21.7 ± 9.3	<0.05
Oncological clearance (%)	93.1	100	NS
Incidence of complications (%)	6.9	15.3	<0.05

### Clinicopathological features of the tumours

There were no significant differences between the tumour location and the gross tumour type in the ESD and radical operation groups (Table 3).

**Table 3:** The pathological type of the patients in the ESD and radical operation groups

	ESD group	Radical operation group	*P*
*n*	29	59	
Highly differentiated adenocarcinoma	17 (58.6)	28 (47.5)	NS
Moderately differentiated adenocarcinoma	9 (31)	19 (32.2)	NS
Minimally differentiated adenocarcinoma	2 (6.9)	7 (11.9)	NS
Signet ring cell carcinoma	1 (3.4)	5 (8.5)	NS

### Comparison of the clinical outcomes between the groups

The median length of the operation was significantly longer for the patients who underwent a radical operation [78 ± 11.4 (65–133) min] compared with ESD [53 ± 7.9 (30–110) min; *P* < 0.05]. The patients treated by radical surgery had a longer median hospital stay [21.7 ± 9.3 (11–55) days] compared with those who underwent ESD [14.3 ± 3.7 (10–23) days; *P* < 0.05]. The median size of the lesions was not significantly different between the two groups (2.7 ± 1.9 vs. 3.5 ± 1.6 cm). There was no perioperative mortality in either group, but the overall complication rate was significantly higher in the radical operation group (Table 2). There was also a significant difference in the depth of invasion between the two groups. The rate of EGC with submucosal invasion was significantly higher in the radical operation group than in the ESD group (*P* < 0.05). The oncological clearance was not significantly different between the two groups (93.1 vs. 100 %, *P* > 0.05). In the ESD group, cancer cells were detected at the margin of the resected lesion in one patient whose pathological type was signet-ring cell carcinoma; adenocarcinoma with an intravascular tumour embolus and a low level of differentiation was detected at the deep margin of the resected lesion in another patient. These two patients underwent another surgical procedure. The incidence of complications in the radical operation group (9/59, 15.3 %) was higher than that in the ESD group (6.9 %, 2/29). In the ESD group, post-ESD delayed haemorrhage and abdominal pain were the major complications. Post-ESD delayed haemorrhage occurred (at 6 and 24 h after ESD) in two patients who presented with hematemesis or/and haematochezia, and both were effectively treated by emergency endoscopic haemostasis. The majority of patients had different degrees of abdominal pain that were relieved after 24–48 h by symptomatic treatment. In the radical operation group, nine patients had complications, including one patient with acute postoperative adhesive ileus, three patients with symptomatic postoperative residual gastritis, one patient with postoperative hypoproteinaemia and seroperitoneum, one patient with postoperative iron deficiency anaemia, two patients with marginal ulcers and one patient with postoperative gastrointestinal disturbance. The patient with acute postoperative adhesive ileus recovered well after re-operation. The other complications were improved or cured by conservative treatment, such as medical treatment, nutritional support therapy or symptomatic treatment (Tables 2 and 4).

**Table 4:** The complications observed in the ESD and radical operation groups

Operation	ESD	Radical operation
Complications		
Postoperative delayed haemorrhage	2	
Perforation		
Acute postoperative adhesive ileus		1
Symptomatic postoperative residual gastritis		3
Postoperative hypoproteinaemia and seroperitoneum		1
Postoperative iron deficiency anaemia		1
Marginal ulcer		2
Postoperative gastrointestinal disturbance		1

### Follow-up in the ESD and radical operation groups

Gastroscopy and observation (using the EORTC QLQ-C30 questionnaire and EORTC QLQ-STO22 questionnaire) were performed at 1, 3, 6, 12, and 24 months post-EDS. All patients completed the follow-up observation. There were no significant differences in the median follow-up period between the ESD [26.9 ± 8.5 months (1–5 years)] and radical operation [22.3 ± 9.4 months (1–3.7 years)] groups. Regular gastroscopy confirmed that the tumours did not recur in either of the groups (Table 2).

### Comparison of the QOL between ESD and radical operation

The overall health status based on the EORTC QLQ-C30 was significantly lower in the radical operation group than in the ESD group (*P* < 0.05). When the 15 functional scales were compared, the physical function and social function categories were significantly lower in the radical operation group (*P* < 0.05); although the role, emotional and cognitive function categories were also lower in the radical operation group than in the ESD group, the differences between the two groups were not significant. In the nine categories pertaining to symptom scales, the only symptoms that reached statistical significance were fatigue (*P* < 0.05), nausea and vomiting (*P* < 0.05), and loss of appetite and constipation (*P* < 0.05), with the symptoms being significantly more common in the radical operation group. The QOL based on all nine categories of the QLQ-STO22 was lower in the radical operation group, but the results were significantly different for only three categories: reflux (*P* < 0.01), eating restrictions (*P* < 0.05) and body image (*P* < 0.05) (Table 5).

**Table 5:** The quality of life of the patients in the ESD and radical operation groups

	ESD group (*n* = 29)	Radical operation group (*n* = 59)	*P*
QLQ-C30 function			
Overall health status	86.8 ± 23.4	76.5 ± 18.6	<0.05
Physical functioning	79.2 ± 16.3	70.5 ± 14.1	<0.05
Role functioning	88.4 ± 25.3	79.8 ± 27.7	NS
Emotional functioning	82.3 ± 18.2	77.5 ± 17.6	NS
Cognitive functioning	87.4 ± 16.2	83.7 ± 15.3	NS
Social functioning	84.9 ± 15.3	72.5 ± 26.8	<0.05
QLQ-C30 symptoms			
Fatigue	15.3 ± 6.5	24.8 ± 11.3	<0.05
Nausea and vomiting	9.6 ± 5.6	17.3 ± 7.8	<0.05
Pain	8.1 ± 3.3	10.8 ± 6.9	NS
Dyspnoea	3.8 ± 2.3	5.5 ± 3.2	NS
Insomnia	9.1 ± 7.5	14.3 ± 5.8	NS
Loss of appetite	7.3 ± 4.2	15.6 ± 8.8	<0.05
Constipation	6.8 ± 5.5	16.2 ± 9.4	<0.05
Diarrhoea	8.5 ± 5.9	11.3 ± 6.3	NS
Financial difficulties	9.9 ± 3.4	13.3 ± 6.5	NS
QLQ-STO22 symptoms			
Dysphagia	10.3 ± 8.7	15.7 ± 7.9	NS
Pain	9.6 ± 5.1	15.3 ± 9.4	NS
Reflux	8.0 ± 8.6	21.5 ± 6.8	<0.01
Eating restrictions	6.7 ± 5.9	13.6 ± 11.8	<0.05
Anxiety	11.9 ± 5.5	18.9 ± 7.1	NS
Dry mouth	11.5 ± 8.9	16.6 ± 6.3	NS
Taste problems	10.3 ± 8.3	14.5 ± 8.9	NS
Body image issues	12.5 ± 5.1	21.4 ± 5.5	<0.05
Hair loss	9.6 ± 3.2	11.6 ± 3.5	NS

*P* were calculated by ANCOVA adjusted for gender and follow up duration

*QLQ* quality of life questionnaire

## Discussion

The therapeutic methods used for EGC can be divided into three categories: endoscopic therapy, laparoscopic-assisted radical resection and standard radical gastrectomy. Anastomotic fistula formation is the most common and serious complication of a radical operation and is one of the main causes of death during the perioperative period. The incidence of anastomotic fistula is 3~6.6 % [19, 20]. Pulmonary infections, pleural effusion, incision infections, abdominal infections, subphrenic infections and gastric paralysis syndrome are also common complications [21–23]. Intraperitoneal haemorrhage, acute myocardial infarction, duodenal stump fistula, gastric dumping syndrome, bile reflux gastritis, anastomotic bleeding, gastric retention, adhesive ileus and anastomotic stenosis are also perioperative and postoperative complications [21–23]. Patients have occasionally died because of postoperative complications. Gastric stump cancer (GSC), iron deficiency anaemia and malnutrition are the most common long-term complications. The incidence of complications for a radical operation for EGC is significantly reduced compared with those for advanced gastric cancer. The 5-year survival rate can be higher than 90 %, especially for intramucosal invasive EGC, which has a 5-year survival rate close to 100 % [24]. A multicentre study with a large sample size reported by Kitano et al. [25] (1294 patients who underwent laparoscopic radical resection of EGC at 16 centres, with a median follow-up of 36 months) indicated that the 5-year tumour-free survival rates after a radical operation for stage Ia and Ib gastric cancer (UICC classification) were 99.8 and 98.7 %, respectively [18]. However, some patients developed different degrees of physiological and nutritional disorders after subtotal gastrectomy, such as iron-deficiency anaemia and malnutrition. Because these can also lead to numerous health problems and a decline in the QOL, they are problems that cannot be ignored.

ESD was first used by Gotoda in 1999 and has been one of the principal methods used for the treatment of EGC and precancerous lesions [26]. It has the advantages of providing a wider local excision, the possibility of obtaining en-bloc specimens with free lateral margins, maintaining the normal physiological structure and allowing an accurate determination of the depth of tumour invasion. A study of ESD for the treatment of early disease at Changhai Hospital in Shanghai indicated that the complete resection rate was 95.2 % and the complete histological clearance rate was 90.2 %. These findings suggest that ESD has similar efficacy to and many advantages over radical surgery because ESD can maintain the normal physiological structure [27, 28].

Bleeding and perforation are the major perioperative complications of ESD [29]. Bleeding is the most common complication, and it frequently occurs in the intraoperative period or during the first 24 h postoperatively. A multicentre study by Goto et al. [30] reported that postoperative haemorrhage occurred in 100 (5.5 %, 62 cases during the first 24 h postoperatively) of their 1184 patients who received ESD gastric mucosal tumours. The incidence of perforations has been reported to range from 3.5 to 6.1 % during ESD. Perforations are divided into macro- and micro-perforations [31]. Micro-perforations are the most common type [31]. Because surgeons can immediately observe and successfully close a perforation with endoclips, these perforations can generally be treated conservatively without the need for emergency surgery [31]. Ono [32] reported that the perforation rate was 3.9–5 %, with postoperative bleeding noted in 7.8–8.7 % of patients in a study of 602 EGC patients undergoing ESD using an insulated-type diathermic knife (IT-ESD), but all were successfully treated using an entirely endoscopic approach without the need for gastrectomy. Luminal stenosis is the main long-term complication of ESD, and endoscopic dilation is an efficacious and safe treatment for this condition [33, 34]. The five-year survival rates for the EGC patients treated by ESD was 96.2–100.0 %, whilst the loco-regional recurrence and distant metastasis rates were approximately 0–17.5 % and were similar to those following radical surgery [35].

The oncological clearance in the present patients in the ESD group was 93.1 % (27/29). Postoperative delayed haemorrhage occurred in two patients. The postoperative QOL was better, and the daily life and the normal work of patients were not significantly affected in this group. In the radical operation group, the oncological clearance was 100 % (59/59). Nine patients had complications, but the symptoms in most patients were markedly improved after multimodality therapy, and very few patients needed reoperation. Some prolonged or recurrent complications, such as postoperative ileus, symptomatic residual gastritis, marginal ulcers and iron deficiency anaemia affected the nutritional status of the patients and negatively impacted their QOL. The EORTC QLQ-STO22 and EORTC QLQ-STO30 also confirmed that the QOL in the radical operation group was significantly lower than that in the ESD group. This is one of the advantages of ESD compared with radical surgery. Gastric stump cancer has not been observed in any of the patients treated by radical surgery. There have also been no cases of loco-regional recurrence or distant metastasis amongst the patients treated by ESD or radical surgery during the follow-up period.

These findings indicate that ESD achieved similar efficacy and was advantageous compared with radical surgery for the treatment of EGC [36]. Nevertheless, only a small number of patients were included in this study, and the median follow-up was not sufficient to evaluate the long-term recurrence rate. Furthermore, it was a retrospective study that may have led to some bias. Although it is theoretically feasible for the patients with EGC to be randomly divided into two groups for ESD or a radical operation, this would not conform to the principles of medical ethics. Along with the ever-increasing use of ESD for EGC and the continuous extension of the follow-up period, we will try to obtain extended follow-up data of more than 15 years in order to evaluate the long-term prognosis of EGC patients treated by ESD and radical surgery. We expect that the results of the present study will be confirmed by prospective randomised analyses and that such studies would provide a more objective evaluation.

## Conclusions

This study indicates that ESD can achieve similar efficacy compared with radical surgery for the treatment of EGC with appropriate case selection. The patients treated by ESD had better outcomes, including a shorter operation, lower incidence of complications, shorter hospital stay, similar oncological clearance and a better QOL than those treated by radical surgery.
